# New Directions for Motivational Incentive Interventions for Smoking Cessation

**DOI:** 10.3389/fdgth.2022.803301

**Published:** 2022-02-28

**Authors:** Lara N. Coughlin, Erin E. Bonar, Maureen A. Walton, Anne C. Fernandez, Isabelle Duguid, Inbal Nahum-Shani

**Affiliations:** ^1^Addiction Center, Department of Psychiatry, University of Michigan, Ann Arbor, MI, United States; ^2^Injury Prevention Center, University of Michigan, Ann Arbor, MI, United States; ^3^Institute for Social Research, University of Michigan, Ann Arbor, MI, United States

**Keywords:** smoking cessation, cigarette, just-in-time adaptive intervention (JITAI), contingency management (CM), motivational incentives

## Abstract

**Background:**

Motivational incentive interventions are highly effective for smoking cessation. Yet, these interventions are not widely available to people who want to quit smoking, in part, due to barriers such as administrative burden, concern about the use of extrinsic reinforcement (i.e., incentives) to improve cessation outcomes, suboptimal intervention engagement, individual burden, and up-front costs.

**Purpose:**

Technological advancements can mitigate some of these barriers. For example, mobile abstinence monitoring and digital, automated incentive delivery have the potential to lower the clinic burden associated with monitoring abstinence and administering incentives while also reducing the frequency of clinic visits. However, to fully realize the potential of digital technologies to deliver motivational incentives it is critical to develop strategies to mitigate longstanding concerns that reliance on extrinsic monetary reinforcement may hamper internal motivation for cessation, improve individual engagement with the intervention, and address scalability limitations due to the up-front cost of monetary incentives. Herein, we describe the state of digitally-delivered motivational incentives. We then build on existing principles for creating just-in-time adaptive interventions to highlight new directions in leveraging digital technology to improve the effectiveness and scalability of motivational incentive interventions.

**Conclusions:**

Technological advancement in abstinence monitoring coupled with digital delivery of reinforcers has made the use of motivational incentives for smoking cessation increasingly feasible. We propose future directions for a new era of motivational incentive interventions that leverage technology to integrate monetary and non-monetary incentives in a way that addresses the changing needs of individuals as they unfold in real-time.

## Introduction

Despite substantial progress in reducing cigarette smoking in the United States, tobacco use continues to be the leading cause of preventable death (1, 2). Most people who smoke cigarettes want to quit, yet barriers to cessation such as limited reach and access to effective treatments and difficulties with user adherence and retention limit successful quit attempts (3). Motivational incentive interventions (MIIs; i.e., incentive-based treatments, contingency management) hold tremendous potential for promoting tobacco cessation. Grounded in operant conditioning theory (4, 5), MIIs facilitate behavior change, such as smoking cessation, through the provision of contingent reinforcers for the desired behavior. Multiple meta-analyses provide robust empirical evidence that clinic-based MIIs are highly effective for treating cigarette smoking and other substance use behaviors (5–7). Yet MIIs remain under-utilized in a large part due to several key barriers. First, the time and effort of administering MIIs in clinics make their implementation highly challenging, hence limiting the feasibility of these interventions (8–10). Second, frequent monitoring and intervention delivery schedules increase burden, reducing the acceptability of MIIs. Third, the up-front costs of providing monetary incentives for abstinence limit the scalability of MIIs. Finally, the literature is still unclear if the exclusive reliance on monetary incentives may limit intrinsic motivation for change (11–14), potentially preventing MIIs from achieving optimal long-term effectiveness following intervention termination.

Increasingly, MIIs are delivered via digital platforms, such as mobile phones and wearables (15). These technologies have the potential to eliminate the need for in-clinic assessment and intervention delivery (16), hence reducing burden, and lowering the cost of frequent abstinence monitoring. However, to fully realize the potential of digital technologies to deliver MIIs it is critical to develop strategies to mitigate concerns that reliance on extrinsic monetary reinforcement may hamper intrinsic motivation for cessation. Moreover, while technology can be used to reduce burden in delivering MIIs, empirical evidence indicates that engagement in digital interventions is suboptimal (17–19). Finally, although technology can be used to reduce some of the costs associated with frequent monitoring, it is beneficial to further address scalability limitations due to the up-front cost of monetary incentives.

The application of new intervention design approaches, such as just-in-time adaptive interventions (JITAIs), may further enhance MIIs feasibility, effectiveness, and scalability. JITAIs leverage mobile and sensing technologies to deliver the right type and amount of intervention, at the right time, while minimizing unnecessary intervention and reducing individual burden (20, 21). In this perspective article, we propose that guidelines and principles for the design of JITAIs can be used to highlight new directions for leveraging digital health tools to deliver MIIs for smoking cessation. To begin, we briefly review the evidence for MIIs for smoking cessation, including recent advances in digital delivery. Then, we review specific guidelines and principles in the design of JITAIs and discuss how they can be employed to enhance MIIs.

## Evolution of the Literature on MIIs for Smoking Cessation

Multiple meta-analyses and systematic reviews support the efficacy of MIIs for smoking cessation (5–7, 22). Emerging evidence shows that digitally-delivered MIIs have similar efficacy to in-person models of delivery (15). These digital methods often use app or web-based platforms to allow for near-instantaneous monitoring and delivery of contingent incentives and to automate intervention delivery. For monitoring of smoking behaviors, digital MIIs often use web or phone cameras and carbon monoxide (CO) devices, through which participants record themselves using the CO device, submitting the video to the study team for simultaneous abstinence and identity verification (15, 22). For example, Motiv8, is a commonly used web and smartphone application in MII smoking cessation studies that allows participants to upload videos and tracks incentive payments (23–32).

MIIs also use digital platforms for instantaneous delivery of monetary incentives for abstinence which can be provided through a variety of methods. Prior studies have used electronic or standard gift cards (24–28, 30, 32–34), mailed checks (35), or funds directly loaded onto a debit card (36). The timing of reinforcement delivery after abstinence verification is critical as the effectiveness of MIIs is nearly twice as large when the incentive is delivered to the participant immediately following verification of the contingent behavior compared to delayed delivery (6, 15, 22). Thus, more rapid incentive delivery methods, such as automatically loading a debit card in the person's possession, are preferable to other slower incentive delivery options (e.g., mailed checks or gift cards). Alternative incentives have also been studied including mailing cessation aids [e.g., nicotine patch, (37)] and using deposit contracts, which involve participants contributing a certain amount of money up-front and earning it back by maintaining abstinence (38). A notable concern about the latter is the potential for limiting treatment access among populations who cannot afford to deposit funds up-front.

Recent advancements in biochemical abstinence verification have the potential to further streamline abstinence monitoring. For example, the iCO device by coVita (https://www.covita.net/) is a pen-sized Bluetooth-enabled breathalyzer used in conjunction with a smartphone that provides real-time cessation verification and facial recognition software to confirm identity. Another example is Autosense (https://sites.google.com/site/autosenseproject/), a suite of sensors in an arm and chest band, interfacing with a smartphone, that is used to passively detect smoking episodes and provide continuous cessation verification (39, 40). Accordingly, as technological advancements such as these continue to develop, barriers to delivering MIIs may be reduced by eliminating the need for clinic-based abstinence monitoring and automating intervention delivery.

**Figure 1:**
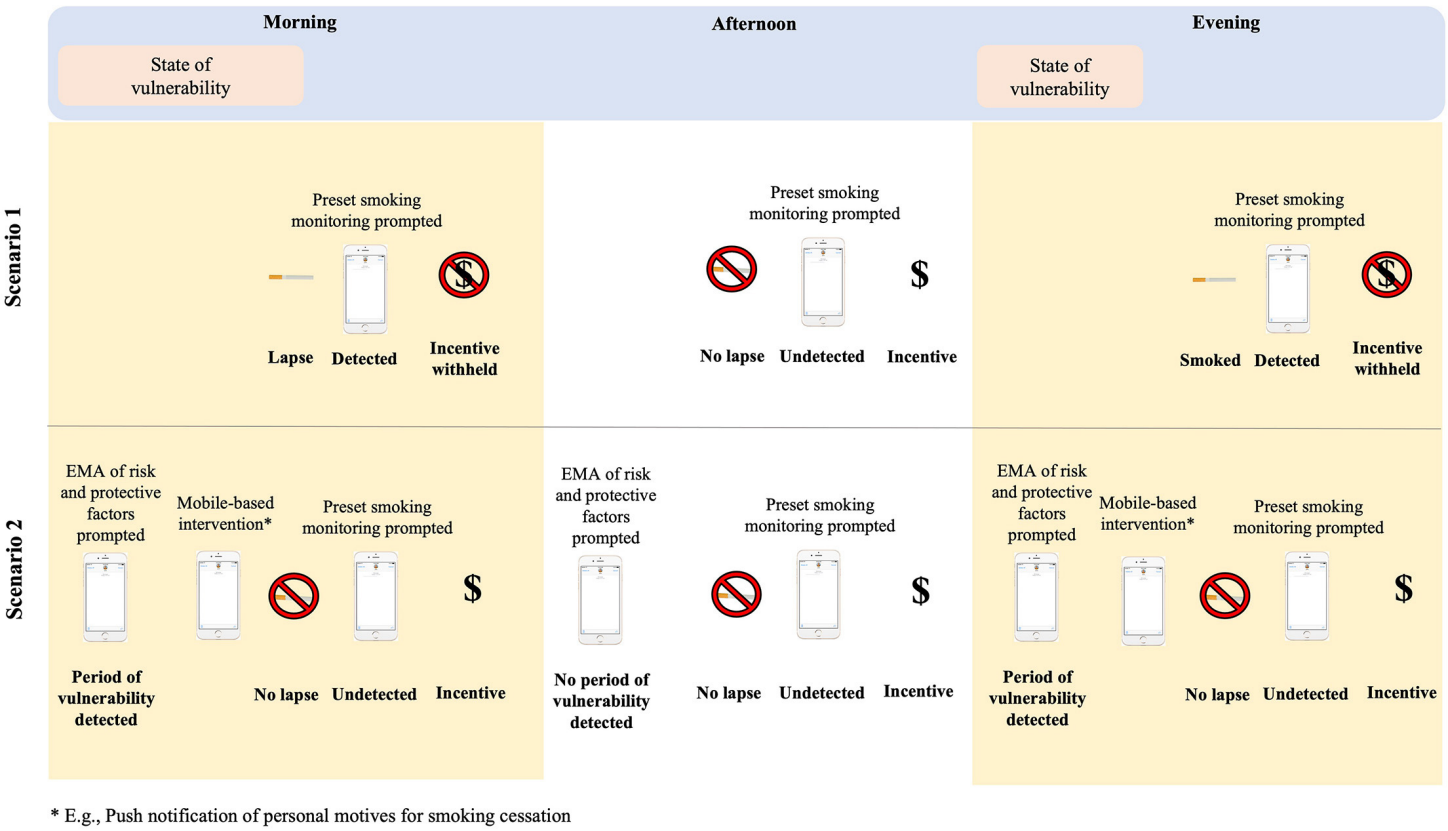
Depiction of conventional digital MIIs (Scenario 1) and a JITAI MII (Scenario 2). EMA, ecological momentary assessment.

Currently, digitally-delivered MIIs have translated intervention procedures from conventional in-person MIIs to digital platforms. Like in-person MIIs, these interventions are adaptive interventions in that they use time-varying information about a person's smoking status (abstinent vs. lapse) to determine if an incentive should be delivered (see Figure 1, Scenario 1 for depiction of general intervention design). Typically, MIIs can monitor abstinence with some frequency (e.g., daily) and deliver a monetary incentive based on this information. The digital evolution of MIIs reduces the burden of in person monitoring and incentive delivery through mobile and wearable technology and digital incentive delivery in close to real-time. To advance digitally-delivered MIIs, it is critical to develop strategies to further reduce the cost associated with reliance on monetary incentives, assess real-time risk for lapse, promote intrinsic motivation for behavior change, and increase participant engagement in digital interventions. JITAIs represent an ideal model to advance MIIs in these areas. JITAIs are adaptive interventions that are designed to address conditions that change rapidly in daily life (20, 21). Several principles in the design of JITAIs have yet to be incorporated in digital MIIs. In the below sections we will discuss these JITAI principles and how they may be leveraged to further increase the feasibility, effectiveness, and scalability of digital MIIs by minimizing cost, facilitating autonomous motivation, and enhancing intervention engagement (see summary in Table 1).

**Table 1:** How JITAI principles can inform a new era of MIIs for smoking cessation.

**JITAI principle**	**In practice**	**Opportunities for mIIs**
Minimize cost	Only deliver an intervention (i.e. incentives) when necessary Offset monetary incentives with non-monetary incentives	Enhance intervention scalability
Deliver the right type of support, at the right time.	Use dynamic information about the person's motivation for change to decide whether and when to deliver monetary vs. non-monetary incentives Leverage information beyond abstinence to address more rapidly changing needs or conditions that can prevent lapse	Enhance the short- and long-term motivation to change and intervention effectiveness
Facilitate sufficient intervention engagement	Avoid unnecessary assessment and intervention: In MIIs abstinence monitoring is part of the intervention; adapt the frequency of monitoring to the person's level of risk (less frequent monitoring if risk is low) Leverage information about the person's state and context to deliver an intervention only when the individual is receptive to (i.e., likely to engage in) the specific intervention Incorporate features (e.g., messages, personalized feedback) to increase receptivity to assessment and intervention content	Enhance the overall effectiveness of the intervention by using information about the person's state to monitor abstinence only when risk of lapse is elevated and incorporate features to increase receptivity to assessment and intervention components

## Applications of Jitai Principles to Advance Digitally-Delivered MIIs

### Principle 1: Minimizing Cost

The scalability and sustainability of MIIs may be limited based on the up-front cost of MIIs to establish and maintain cessation over time (41, 42), especially in the absence of wide scale insurance coverage for treatment (43, 44). The cost and cost-effectiveness of MIIs varies across intervention protocols, populations, and magnitudes of monetary incentives (45–48). However, a consistent finding is that up-front costs for MIIs, including digitally-delivered MIIs, remain high, with monetary incentives often exceeding $500 per person (32, 36, 49). An important principle in the formulation of JITAIs is the delivery of interventions, in this case monetary incentives, only when they are needed. This principle has the potential to reduce the overall cost of MIIs by reserving monetary incentives for those conditions in which they are necessary to maintain abstinence and providing non-monetary incentives or other intervention content when monetary incentives are not necessary to meet treatment goals.

For example, the case may be that non-monetary incentives, such as verbal reinforcement (“Great work!”) or app-based certificates of achievement (e.g., tokens), are sufficient when risk for smoking is low, such as when people report minimal craving to smoke or high confidence in their ability to maintain cessation. Conversely, non-monetary incentives may not be as effective during higher risk periods for lapse indicating a need to preserve monetary incentives for these instances. As another example, over the longer-term course of treatment (e.g., weeks, months), information about patterns of use (e.g., percent of abstinent breath CO samples) may be used to modify the intensity and type of support delivered and to inform when the person has received an adequate dose of treatment (50, 51). As risk for lapse reduces, MIIs may shift toward delivering a larger proportion of non-monetary reinforcers and a smaller proportion of monetary incentives. Future evaluation of the ongoing estimated likelihood of maintaining cessation (e.g., based on information about percent of recent abstinent CO samples) could inform points in treatment where a step down in care is indicated. Here, operant conditioning principles such as fading [i.e., reducing the magnitude or frequency of monetary incentives; (52)], which are already common in MIIs [e.g., (27, 33)], could be deployed based on the individual's estimated likelihood of maintaining cessation to ensure that only the minimum necessary monetary incentives are provided. We will discuss considerations for identifying when different types of intervention are optimal in Section Principle 2: Deliver the Right Type of Support, at the Right Time below.

### Principle 2: Deliver the Right Type of Support, at the Right Time

Perhaps the most frequent concern about MIIs is that people often return to smoking when the contingency (e.g., monetary incentives) is removed, limiting the durability of behavior change (44, 53). Although a recent meta-analysis found that sustained, long-term abstinence following MIIs for substance use disorders in general is comparable or exceeds other gold standard treatments (e.g., cognitive behavioral therapy), rates of relapse across all treatments remain all too common (54). Application of JITAI principles to optimize the type and timing of MII support may improve the overall (i.e., short- and long-term) effectiveness. Currently, MIIs typically focus on a single contingent behavior, such as CO breath samples, requested at a fixed interval (e.g., twice daily) to determine if the intervention, usually a monetary incentive, will be delivered. Augmentations to existing MIIs to optimize the type and timing of support to be delivered may take several different forms. To better detect times when intervention support is needed, MIIs may incorporate other tailoring variables (in addition to abstinence monitoring), such as factors to detect increased risk for lapse in the near-term (e.g., craving, self-efficacy). MIIs may also consider varying reinforcement schedules, including adaptations to the frequency of abstinence monitoring, magnitude of incentives based on time-varying risk factors for smoking lapse, or type of incentives delivered (e.g., monetary or non-monetary incentives). In the next paragraphs, we will review possible adaptations to monitoring and reinforcement schedules in MIIs, with the goal of delivering the right type of support at the right time to improve short- and long-term outcomes. One way that JITAIs can inform when intervention is needed is by identifying states of vulnerability to an adverse outcome and states of opportunity for positive behavior change. States of vulnerability [i.e. periods of heightened susceptibility to smoking lapse (21)], often emerge rapidly and may indicate a need for just-in-time support to prevent lapse. Identification and incorporation of vulnerable states is yet to be applied within the context of digital MIIs. Current MII designs provide monitoring of abstinence, and incentives are typically contingent on abstinence verification. Recently, Businelle et al. (55) were able to identify periods of vulnerability for near-term (within 4 h) smoking lapse using ecological momentary assessments (EMAs) of common risk factors for lapse (e.g., craving, stress, alcohol consumption, motivation, interaction with someone smoking, and cigarette availability). If similar monitoring of risk factors were incorporated into MIIs, then during states of vulnerability, incentives (monetary or non-monetary) could be delivered to potentially break the link between periods of heightened risk and smoking lapse, thereby increasing the chances of maintaining cessation.

Figure 1 provides an illustrative example of how the JITAI principle to provide the right type of support, at the right time, can be operationalized in MIIs by identifying states of vulnerability to smoking lapse and adapting the delivery of incentives based on this information. Figure 1 contrasts two hypothetical scenarios for illustration—Scenario 1 which includes a conventional digitally-delivered MIIs for smoking cessation, and Scenario 2 which includes a digitally-delivered MII that incorporates JITAI principles. In Scenario 1, states of vulnerability to lapse are not detected or intervened upon. Accordingly, when the person experiences states of vulnerability in real-world settings, these states are likely to lead to a smoking lapse and thus to withholding the contingent incentives. In Scenario 2, where states of vulnerability are monitored via EMAs, a just-in-time mobile-based intervention (e.g., push notification about the person's motive for cessation) may provide the necessary support to maintain cessation through the period of heightened risk. Future work is needed to determine how to identify states of vulnerability (e.g., via geolocation, self-report EMAs of risk factors) and how to best intervene to prevent a lapse. Furthermore, future work may also evaluate if, in some instances, non-monetary incentives provide sufficient reinforcement.

In contrast to periods of vulnerability, periods of opportunity are marked by susceptibility to *positive* behavior change to maintain cessation. Predictors of these periods may include objective measures of continuous smoking cessation, subjective factors such as self-reported self-efficacy or motivation for change (56, 57), and more stable factors (e.g., personality factors, social support). During states of opportunity, JITAIs may capitalize on improved self-efficacy in one's ability to maintain cessation and consider use of non-monetary incentives or of offering an alternative monetary incentive with the goal of enhancing incentive salience while possibly also reducing costs. Options for alternative monetary incentive schedules that are already established in the MII field include deposit contracts (i.e. where previously earned incentives are committed up-front and can be recouped for a specified period of abstinence, leveraging loss aversion) or changing the incentive schedule from a smaller, fixed-rate incentive to a larger, probabilistic incentive schedule (e.g., such as 25/75 odds of larger or no incentive, which targets availability bias). These incentive schedules may be offered to people during times of relative lower risk for smoking as a way to both increase novelty and potentially decrease cost. Alternatively, identification of states of opportunity may, in some cases, indicate that no intervention is needed as treatment gains are already likely to be maintained.

When considering the right type and timing of intervention delivery, another potential avenue for MIIs is to identify when different types of reinforcers are most effective. Monetary incentives are typically used in MIIs and a common concern is that monetary incentives promote external regulation of behaviors (i.e. reinforcement is contingent on a behavior such as evidence of maintained cessation), which is the least autonomous form of motivation (58). Notably, prior investigations have reported counterintuitive effects of MIIs on motivation and self-efficacy for behavior change, with some indications of no difference or increases in these constructs compared to control conditions (11–14). However, investigators also note the need to improve and sustain autonomous motivation to facilitate sustained behavior change, particularly once the intervention ends (13, 59).

The singular focus of MIIs on overt, external monetary incentives may limit autonomous motivation for smoking cessation (60), potentially hampering sustained cessation. Here, autonomous motivation is defined as the internalization and integration of the value of activities [e.g., smoking cessation; (59)]. Accordingly, the integration of non-monetary extrinsic reinforcement that supports greater autonomy alongside conventional monetary incentives has the potential to improve longer-term motivation for behavior change and outcomes. Indeed, prior work in adolescents and young adults who use alcohol or cannabis found that personalized information messages, which they considered to be non-monetary incentives, increased engagement with daily self-report measures in some circumstances, particularly when the person was at higher risk of disengagement (19). Non-monetary incentives to promote sustained motivation in digital MIIs may include a variety of psychologically-based incentive options to foster intervention adherence and abstinence (61), such as rewards (e.g., tokens) for behavior change milestones, provision of desired information (e.g., personalized information), or praise for positive behavior change. A key future step to incorporate non-monetary incentives into MIIs is to determine when these incentives are effective and when monetary incentives are still most useful. Scientific investigation to determine when monetary or non-monetary incentives are most effective, and balancing this with the principle of minimizing cost (see Section Principle 1: Minimizing Cost), may improve both the long-term effectiveness and scalability of MIIs.

### Principle 3: Facilitate Sufficient Engagement

Digital delivery of MIIs has the potential to reduce individual burden by eliminating the need for frequent in-clinic visits for abstinence monitoring. Yet, burden is still an issue even when technology is used due to the tremendous amount of information individuals typically receive from digital devices in everyday life. The added burden of digitally-delivered MIIs in addition to the existing standard information load may undermine engagement in digital assessments and intervention content. An important principle in the formulation of JITAIs is to facilitate sufficient engagement (i.e., the extent and duration of engagement in the intervention needed to achieve a pre-specified distal outcome). This can be achieved not only by delivering the right intervention at the right time, thereby enhancing the self-relevance of the intervention; (62–64), but also avoiding the initiation of assessments and the delivery of interventions that are not necessary for achieving the pre-specified long-term goal of the intervention.

Abstinence monitoring is a central component of MIIs, with abstinence verification often requested frequently (e.g., daily or more) to determine incentive delivery. A lack of engagement-promoting features has limited MIIs from meeting their full potential, with reports of low engagement (e.g., <50% adherence to cessation monitoring) limiting intervention feasibility and overall effectiveness (27, 30, 65–67). JITAIs are explicitly designed to provide interventions when people are receptive to the intervention, that is when an individual is willing and able to receive, process, and use the specific just-in-time intervention (20, 21). For example, people may be more likely to engage with abstinence monitoring prompts if they are delivered in particular contexts, such as when they are alone or at home, or at particular times of day, such as during a lunch break or right after work. Furthermore, prior work leveraging reciprocity, or people's innate psychological and normative tendency to return favors (68), as a means of encouraging engagement shows that sending non-contingent, unsolicited content (such as an inspirational quote) prior to prompting app engagement increases the likelihood of adherence, especially on days of the week where engagement is more likely (19). In recent work by this study team, app users who drank alcohol or used cannabis suggested provision of more personalized feedback, such as graphs showing tracking of behaviors over time (e.g., substance use, stress), as a way to increase app appeal (69). Indeed, personalized feedback about abstinence progress is already provided in some digital MIIs, though it is not typically evaluated for the independent influence on treatment outcomes (15). To the extent personalized feedback about abstinence and other monitored states (e.g., craving, stress) is also appealing to those who smoke cigarettes, these features can continue to be incorporated and expanded within the digital MII environment. Accordingly, the addition of engagement-promoting digital health features may help to improve engagement in MII monitoring components by prompting engagement when people are likely to be receptive.

An implicit assumption of MIIs is that an individual should be rewarded when abstinent. Thus, in conventional MIIs incentives are delivered consistently following abstinence verification. However, in some cases this assumption may not hold. For example, it is possible that the benefits of contingent monetary incentives dissipate over time due to habituation, necessitating the delivery of other types of incentives to enhance attention, interest, and anticipation of the person. JITAIs can be employed to collect information beyond abstinence to inform whether and what type of incentive is most needed to promote participant engagement. Furthermore, JITAIs can also inform when abstinence monitoring is necessary based on risk of lapse. For example, during periods of high-risk of lapse (e.g., soon after an initial quit attempt or in a high-risk context such as around other people who are smoking) frequent monitoring may be necessary. However, some periods of vulnerability may be marked by reduced receptivity to monitoring and intervention. JITAIs may also be used to identify lower risk periods where less frequent abstinence verification can reduce burden without sacrificing effectiveness. More empirical evidence is needed to determine whether and how it is best to intervene when individuals are at higher and lower risks for lapse.

## Discussion

Frequent and low-burden monitoring of behaviors, delivery of real-time interventions, and rapid assessment of intervention responses may mark a new age in smoking cessation treatments. Guided by JITAI principles, digital MIIs have the potential to achieve greater effectiveness and scalability, opening the door for advancements in the current science of motivational incentives for smoking cessation. Use of digital health technology can enable the identification of states of vulnerability to lapse (e.g., urge to smoke, presence of other smokers) and opportunities to fortify gains (e.g., maintain cessation through building motivation and self-efficacy) and inform when and what type of incentive (or lack thereof) will be most helpful. Specific to MIIs, the integration of monetary and non-monetary reinforcers, and identification of specific conditions in which different types of incentives are most effective, may have the dual benefit of increasing autonomous motivation for behavior change and also reducing up-front costs of exclusive reliance of monetary incentives. Finally, the incorporation of engagement-promoting features can enhance adherence to JITAI-related assessments and interventions, potentially improving the overall effectiveness of the MII. Thus, MIIs using JITAIs to deliver both monetary and non-monetary incentives, and incorporating engagement-promoting features, have the potential to fill the significant gap that exists between the current state of MIIs and a potential new wave of more engaging and durable interventions promoting sustained smoking cessation.

The next steps for the proposed line of work include applying established interactive digital health development frameworks (70) to existing MIIs to create a new wave of MIIs based on JITAI principles. These new interventions—building on the rich foundation of MIIs (8, 9, 15, 54) and integrating it with what is known about the design of JITAIs (17–19)—have the potential to enhance the ability of MIIs to affect long term smoking cessation. In summary, digitally-delivered MIIs can benefit from employing JITAI principles by adapting incentive structures, integrating non-monetary reinforcers, and identifying when people are most receptive to intervention to meet the rapidly changing needs of those who are attempting to quit smoking.

## Data Availability Statement

The original contributions presented in the study are included in the article/supplementary material, further inquiries can be directed to the corresponding author.

## Author Contributions

LC conceived of and wrote the first draft of the manuscript. ID and IN-S wrote sections of the manuscript. All authors contributed to manuscript revision, read, and approved the submitted version.

## Funding

LC's time was funded through NIAAA K23 AA028232. IN-S support included U01CA229437, P50 DA054039, and R01 DA039901.

## Conflict of Interest

The authors declare that the research was conducted in the absence of any commercial or financial relationships that could be construed as a potential conflict of interest.

## Publisher's Note

All claims expressed in this article are solely those of the authors and do not necessarily represent those of their affiliated organizations, or those of the publisher, the editors and the reviewers. Any product that may be evaluated in this article, or claim that may be made by its manufacturer, is not guaranteed or endorsed by the publisher.
